# Hierarchy of Idea-Guided Action and Perception-Guided Movement

**DOI:** 10.3389/fpsyg.2012.00579

**Published:** 2012-12-27

**Authors:** Sasha Ondobaka, Harold Bekkering

**Affiliations:** ^1^Donders Institute for Brain, Cognition and Behaviour, Radboud UniversityNijmegen, Netherlands

**Keywords:** voluntary behavior, goal-hierarchy, ideomotor principle, idea-guided action

## Abstract

The ideomotor theory of voluntary behavior assumes that the selection and control of a concrete goal-directed movement depends on imagining its direct perceptual consequences. However, this perception-guided assumption neglects the fact that behavioral control entails a hierarchical mechanism wherein conceptual expectations – action goals – can modulate lower level perceptuo-motor representations. In this paper, we focus on the hierarchical nature of voluntary behavior by distinguishing between perceptual representations of *images* that relate to attainment of concrete movement goals and conceptual representations of *ideas* that pertain to attainment of action goals. We review the dominant ideomotor principle of the direct perceptuo-motor relation and examine its limitation in the light of the proposed hierarchical view of voluntary behavior. Finally, we offer a revision of the ideomotor principle that allows extension of its explanatory domain from perception-guided movement to conceptual, idea-guided action.

In his seminal work “The principles of psychology,” William James popularized the notion that all voluntary behavior entails a fundamental principle of ideomotor action (James, 1890). The ideomotor principle states that selection and control of a particular movement depends on the anticipation of a sensory effect, which is normally experienced as its immediate product. This notion has been inherited by modern-day empirical psychology and has been translated into the experimental prediction that movement execution should be influenced by a perceptual image of the movement’s effect. Proponents of the ideomotor principle have repeatedly employed stimulus-response paradigms to demonstrate that simple movements are directly linked to the perception of their effects. This work resulted in a plethora of reports in support of the prediction of “perception-guided” movement selection (Greenwald, 1970a; Chartrand and Bargh, 1999; Brass et al., 2000, 2001; Kunde, 2001; Kilner et al., 2003; Pfister et al., 2010), making the perception-guided ideomotor principle fundamental to various accounts of voluntary behavior (Greenwald, 1970b; Prinz, 1987; Hommel et al., 2001; Haggard, 2005; Kunde et al., 2007; Custers and Aarts, 2010).

In everyday goal-directed behavior, however, actors do not typically voluntarily decide which concrete movements to execute. Instead, decisions to move are contingent on the expectations regarding actor’s higher-order, conceptual goal (Jacob and Jeannerod, 2005; Adolphs, 2009; Hauser and Wood, 2010). For example, guidance of everyday voluntary behavior, like getting in touch with a friend, entails both the expectation of the conceptual goal – choosing to make a phone call (or to send an email by using the laptop), as well as selection of concrete movement goals, for example grasping the phone (Searle, 1983; Mele, 1992; Grafton and de Hamilton, 2007; Kilner et al., 2007; Pacherie, 2008). Present versions of the ideomotor principle are not well suited to provide an understanding of these more complex real life behaviors. Understanding the mechanisms that underpin guidance of more complex behaviors requires explanation of the role of prior conceptual knowledge (Johnson-Frey, 2003; Jacob and Jeannerod, 2005), over and above the anticipation of movement’s sensory consequences.

Whereas ample evidence supports “perception-guided” movement, at present few experiments have investigated the role of conceptual goals in selection and control of behavior. There are several reasons why this could be the case. First, the practical difficulties of empirically examining how individuals’ conceptual goals influence their behavior are clear. Conceptual goals relate to actor’s internal states that cannot be directly perceived by the observer’s senses, but need to be inferred by recruiting their own conceptual knowledge to make sense of the observed behavior. Second, theories that are tailored to explain voluntary behavior often implicitly adopt versions of behaviorist (perceptuo-motor) principles, which inherently do not include the involvement of conceptual representations in explanations of guidance of voluntary behavior (Lashley, 1951; Jacob and Jeannerod, 2005). In this article we will review some new studies that investigated the role of higher-level conceptual action goals in selection and control of one’s own behavior and in processing of other individuals’ observed behavior. We conclude with a proposal for a revision of the ideomotor principle that allows extension of its explanatory domain from the perceptuo-motor level of perception-guided movement to the conceptual level of “idea-guided action.”

## Perception-Guided Voluntary Behavior

Paradoxically, the term ideomotor action was first coined by the British physiologist William B. Carpenter (1852) to explain peculiar involuntary movements that are executed by individuals independent of their conscious action intention (for a historical review, see Stock and Stock, 2004). Later, the ideomotor action gained ground as the fundamental principle for the account of voluntary behavior (James, 1890). Carpenter, like James, was dedicated to demystifying the underlying neurocognitive mechanisms of movements, which can be observed in a variety of “psychic” phenomena (e.g., Ouija boards, moving tables, the divining rod, the magic pendulum). To provide a rational explanation for these peculiar phenomena, Carpenter and James proposed a principle of ideomotor action that assumes a direct link between perception and movement. Furthermore, in addition to an anticipation of the sensation of movement effects, ideomotor action is proposed to require a presence of a conceptual expectation (an idea) that a certain action will occur (Carpenter, 1852). In other words, hand movement over the Ouija board should be contingent to the idea (expectation) that some “psychic” force will generate movement. However, present day research on the ideomotor phenomena has mainly neglected the role of more abstract expectations in guidance of goal-directed movements.

More recently, selection and control of involuntary movements caused by the direct perception of similar movements of other individuals (i.e., mimicking) has received substantial interest from researchers in psychology (e.g., Prinz, 1997; Dijksterhuis and Bargh, 2001; Hommel et al., 2001). For example, Wofgang Prinz and colleagues (Prinz, 1987, 1997; Hommel et al., 2001) proposed that perception, planning, and control of movements share a common representational domain. To find support for common representations of perception and movement, participants executed finger-movements in response to an arbitrary number while observing task-irrelevant images of movements, similar or dissimilar in terms of the movement direction (e.g., Brass et al., 2000; Brass et al., 2001). The findings showed that movements that were similar to the perceived image were executed faster, compared to the dissimilar ones; providing evidence for a direct “perception-guided” movement.

In a similar vein, an account of social behavior put forward by social psychologists Dijksterhuis and Bargh (2001) introduced the elegant notion of the “perception – movement expressway” to explain that people tend to copy the observed behaviors of other individuals. The core notion put forth by the authors is that people have a natural tendency to imitate their conspecifics, which in some social settings need to be inhibited in order to carry out volitional action. For example, Chartrand and Bargh (1999) have demonstrated that individuals copy movements of the coactors with whom they interact, without the presence of a conscious action intention to do so. These authors instructed participants to rate photographs together with a confederate coactor who either repeatedly shook their foot or rubbed their face. The results showed that participants shook their foot more often while working with the confederate who shook their foot and rubbed their face more often when they perceived face rubbing. This notion that movement execution is automatically governed by perception of similar movement is in agreement with the ideomotor proposal of “perception-guided” movement.

The neurocognitive mechanism that was proposed to account for the reported social and cognitive psychology findings relies on a direct coupling/common representation of observed and executed movement (Prinz, 1997; Hommel et al., 2001; Hommel, 2009). More recently, a useful distinction has been made between the “weak” ideomotor principle that entails an intermediate step between sensory prediction and movement execution and “strong” ideomotor principle, which assume no cognitive intermediation (Shin et al., 2010). For example, some authors consider current ideomotor theory to be “weak” due to the apparent perception-action duality that is maintained (Richardson and Michaels, 2001). Notably, although ideomotor accounts of voluntary behavior emphasize the role of abstract ideas (i.e., concepts) that represent action expectation, the empirical work is mainly focused on the associative mechanisms that underpin perception-guided movement. The bias toward the perception-movement coupling in behavioral control has led to the formation of associative perception-guided theories of voluntary behavior. These theories have mainly neglected the role of abstract ideas (i.e., expectations) that are present prior to any association of perception and movement (Lashley, 1951) and have been criticized for a number of reasons. For example, the theories are deemed limited in their focus on arbitrary perceptuo-motor mappings (i.e., simple button presses to arbitrary stimuli – associating left perceptual feature with a left motor response). Therefore, ideomotor theories are still limited in their capability of explaining everyday object-related action, which necessitates conceptual knowledge regarding functional properties of the used objects (e.g., Johnson-Frey, 2003).

## Idea-Guided Voluntary Behavior

Following reasoning derived mainly from introspection (Lotze, 1852; James, 1890), research on voluntary behavior in cognitive and social psychology focused on the mechanism of direct coupling between perception and movement (Greenwald, 1970b; Prinz, 1997; Dijksterhuis and Bargh, 2001; Hommel et al., 2001), stripped away from the higher, conceptual levels of action control (Lashley, 1951). In contrast, recent theoretical and computational work proposes that the control and planning of simple bodily goal-directed movements depends on prior conceptual expectations that are related to achievement of a particular outcome (Searle, 1983; Wolpert et al., 2003; Grafton and de Hamilton, 2007; Kilner et al., 2007; Pacherie, 2008). For example, it has recently been proposed that a multitiered model underpins selection and control of one’s own behavior (Grafton and de Hamilton, 2007), as well as predicting and understanding the behavior of other individuals (Kilner et al., 2007). Grafton and de Hamilton (2007) proposed a hierarchy of control which includes: (1) the conceptual level of action intention, (2) the concrete movement goal level needed to realize the intention, (3) motor commands that activate the muscles to attain the movement goal, and (4) body kinematics that entail a synergy of different muscles to produce movements in time and space. Also, a recently proposed active inference account suggests that a hierarchy of predictions underpins both observation and execution of movement, without distinguishing between sensory and motor representations (Clark, in press; Friston et al., 2012).

This seemingly opposing nature of the perceptuo-motor (associative) and conceptual (hierarchical) approaches of action control has created tension and opacity throughout the fields of psychology and cognitive sciences. In order to relieve the tension created between the perceptuo-motor and conceptual views of voluntary behavior, the field needs to focus on the interplay between prior conceptual knowledge about the world and the perceptuo-motor associations that are formed by our experiences (Ochipa et al., 1992; Hodges et al., 2000; Wolpert et al., 2003; Pacherie, 2008; Adolphs, 2009). For example, a strong line of evidence from neuropsychology suggests that selection and control of goal-directed behavior entails involvement of a conceptual system that includes world knowledge about objects and their abstract properties and a perceptuo-motor production system that includes information regarding object manipulation (Roy and Square, 1985; Ochipa et al., 1992). Crucially, Ochipa et al. (1992) showed that a damaged conceptual system causes impairments related to abstract knowledge about objects, referred to as conceptual or ideational apraxia, whereas damage to the perceptuo-motor system leads to ideomotor apraxia – impairments of concrete movement production (Roy and Square, 1985).

Carpenter (1852) has already pointed out that perception-guided movement is contingent on the conceptual expectation that a particular movement will occur. It is important to note that Carpenter’s suggestion that multiple levels might be involved in selection and control of behavior resonates with the hierarchical accounts of action control (Lashley, 1951; Searle, 1983; Roy and Square, 1985; Grafton and de Hamilton, 2007; Kilner et al., 2007; Pacherie, 2008). Nevertheless, most psychological experiments throughout the last decades, even those investigating the ideomotor action (e.g., Brass et al., 2000), have neglected this notion of apparent hierarchy. Various paradigms had a limited focus on the relation between the execution of simple movements and the perceptual images that they produce. Important to note is that even though many experiments try to avoid addressing the conceptual level of action control, it is inevitably present in the participants’ explicit or implicit expectations during selection and guidance of their voluntary behavior (Clark, in press).

## The Hierarchy from Perception-Guided Movement to Idea-Guided Action

A number of recent experimental paradigms were employed to investigate the role of conceptual expectations that pertain to abstract ideas in governing selection and control of goal-directed movements (Massen and Prinz, 2007a,b; Liepelt et al., 2008; Ondobaka et al., 2011). For example, in a study by Ondobaka et al. (2011) participants were facing a coactor while sitting at the table with an integrated touchscreen on which, on each trial, four playing cards appeared – one in each corner. First, the coactor selected the higher or lower card of the two cards that were revealed in front of him. This action then led to the immediate revelation of the participant’s cards. Participants were instructed to either match or mismatch the coactor’s conceptual goal (i.e., to select the higher or lower card). During the experiment, regardless of the task (matching or mismatching the conceptual goal), participants received trials in which their response led to matched or mismatched movements with the coactor. For example, in the “match conceptual goal” case, on some trials, the conceptual goal (e.g., the higher card) required the same movement to be carried out by both participants and coactor (e.g., reach to the left). In other trials, the same conceptual goal match may lead to a mismatch of movement goals (e.g., the coactor reaches right for the higher card and the participant must then reach left for the higher card). Findings indicated that participants’ movement execution was solely influenced by the perception of the coactor’s movement (i.e., left or right) when their conceptual goals also matched the coactor’s, suggesting that a hierarchy of intentions governs goal-directed behavior. That is, conceptual goals apparently sit atop the perception-guided level in the hierarchical control of overt behavior.

Interestingly, a similar hierarchy between perception-guided and idea-guided action control has been investigated in tool-use (Massen and Prinz, 2007a,b, 2009). In a series of experiments, the authors employed a joint tool-use paradigm wherein two participants, in consecutive order, touched one of two targets. Participants could touch the target by moving a lever that could translate around one of two activated pivotal points, resulting in the opposite movement-to-target action rule. The setup allowed the authors to manipulate congruency between coactors’ kinematics (moving toward or away from the body), concrete movement goals (target location) and abstract action goals (action rules indicating target-to-movement mapping) in order to test which goal level most strongly influenced observers’ performance. The results suggest that observer’s performance accuracy was significantly higher when abstract action goals (rules) matched, compared to condition in which coactors’ action goals mismatched. Moreover, Massen and Prinz (2009) reported that only when the two coactors adopted the same action goal (idea-guided action), was an effect of match in kinematics and movement goals (perception-guided movement) observed. In contrast, when their action goals mismatched, no perception-guided movement effect was observed. These results are in line with findings from the social card-selection study (Ondobaka et al., 2011) and indicate a guiding role of expectation regarding conceptual goals in the processing of concrete movement goals.

## Hierarchical Ideomotor Account of Voluntary Behavior

**Figure 1:**
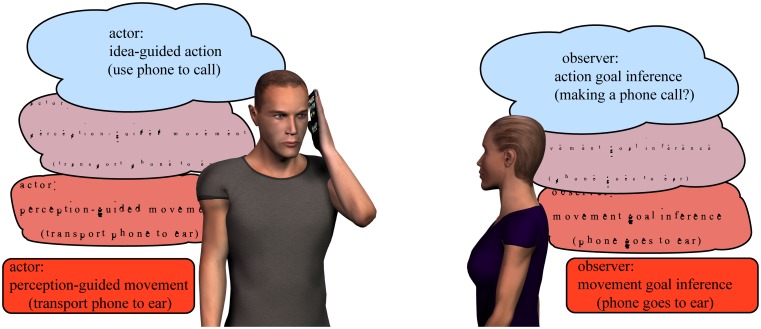
**Illustration of a hierarchical ideomotor principle of action in execution and observation**. The actor generates action expectations (i.e., ideas) at the abstract conceptual level (action goal to make a phone call) as well as at the movement level (movement goal to transport the phone to the ear) and infers these by evaluating the sensory inputs. The observer needs to infer both aspects as well. First, is the actor going to make a phone call? This conceptual goal inference depends on recruiting the conceptual knowledge regarding the function of the object. Second, is the actor bringing the phone to the ear? This movement goal inference depends on the concrete perceptuo-motor associations that link phone to the ear.

Results from these studies are in congruence with Carpenter’s original proposal of the ideomotor action principle, which goes beyond perception-guided movement and stresses the importance of conceptual action expectation in the guidance of voluntary behavior. Recent findings show that in the social contexts wherein abstract ideas govern voluntary behavior, perception-guided effects are present only if coactors’ ideas match (Massen and Prinz, 2009; Ondobaka et al., 2011). Likewise, findings are in agreement with the assumption that the selection and control of one’s own voluntary goal-directed behavior entails a hierarchical mechanism wherein expectation of conceptual goals can modulate concrete bodily movements in space and time (Figure 1). For example, engaging in a goal-directed behavior like getting in touch with a friend entails idea-guided conceptual goal to make a phone call. Subsequently, selection and control of concrete image-guided movements involved in grasping the phone transporting it to the ear must depend on the expectation that the phone will be used in the first place (and not the laptop) and results in the inference of one’s own behavior as getting in touch with a friend. Similarly, understanding whether the observed actor is getting in touch with a friend requires a parallel inference of both conceptual and movement goals (Kilner et al., 2007) by relying on the same hierarchical mechanism that is used for action execution in the observer (Figure 1).

The current proposal asks for an extension of the dominant approaches of voluntary behavior (Greenwald, 1970b; Hommel et al., 2001; Heyes, 2011), which mainly focus on the direct perception-movement links that underpin the generation of perception-guided movement. Consequently, we suggest that dominant views on the nature of perception-action coupling (Greenwald, 1970b; Hommel et al., 2001; Heyes, 2011) should be extended to allow the influence of idea-guided action on top of perception-guided movement (Lashley, 1951; Oztop et al., 2005; Grafton and de Hamilton, 2007). Following the active inference account (Clark, in press), the current proposal can be viewed as a strong version of the ideomotor account – a version that does not necessitate any intermediate cognitive steps in order to translate perceived input into movement. Our hierarchical ideomotor framework states that bodily movement fulfills conceptually guided proprioceptive and visual expectations, without the necessity of an intermediate cognitive process. However, the proposed action hierarchy implies that an antecedent state of expectation does play a fundamental role in shaping perception and action. Crucially, the addition of the conceptual-perceptual (i.e., idea-image) hierarchy and the extension of the anticipatory ideomotor mechanism to the conceptual level leads to significant theoretical advances. First, the incorporation of the conceptual level (Johnson-Frey, 2003) allows the current account to explain everyday object-related action. Second, the proposal maintains the indistinguishable nature of sensory and motor representations, but allows prior expectations to play a modulatory role in the anticipation of sensory consequences that are directly related to movement execution.

Collectively, we summarized recent studies that demonstrate the fundamental role of idea-guided behavior (Massen and Prinz, 2007a,b; Ondobaka et al., 2011) and proposed an extension of the ideomotor principle’s explanatory domain from perception-guided movement to conceptual, idea-guided action. The proactive and hierarchical nature of the extensions accommodate the pivotal role of prior conceptual expectation (i.e., ideas) in providing a scaffold for direct perceptuo-motor coupling, thus maintaining the “strong” ideomotor character. At the same time, the proposal is in accordance with the origins of the ideomotor principle (Carpenter, 1852), in which a fundamental role for prior expectations in voluntary movement is already suggested. Adopting the hierarchical ideomotor view wherein action concepts and movement goals interact during selection and control of action could potentially unify the perceptuo-motor and conceptual frameworks of voluntary behavior.

## Conflict of Interest Statement

The authors declare that the research was conducted in the absence of any commercial or financial relationships that could be construed as a potential conflict of interest.
